# Complete Genome Sequences of Three Salmonella Strains Obtained from a Poultry Production Farm in Thailand

**DOI:** 10.1128/mra.01104-22

**Published:** 2023-01-18

**Authors:** Tantip Arigul, Piroon Jenjaroenpun, Natnicha Wankaew, Juthatip Vespirom, Phatcharaporn Thuamkaew, Thidathip Wongsurawat, Sudarat Ledlod

**Affiliations:** a Division of Medical Bioinformatics, Research Department, Faculty of Medicine Siriraj Hospital, Mahidol University, Bangkok, Thailand; b Siriraj Long-Read Lab (Si-LoL), Faculty of Medicine Siriraj Hospital, Mahidol University, Bangkok, Thailand; c Laboratory Department, CPF (Thailand), Kangkoi, Saraburi, Thailand; d Research Department, CPF Food Research and Development Center Co., Ltd., Lamsai, Wangnoi, Phranakhon Si-Ayutthaya, Thailand; University of Maryland School of Medicine

## Abstract

Here, we report three complete circular genome sequences of Salmonella enterica SalSpp07, SalSpp08, and SalSpp09, which were isolated from chicken meat and skin during quality control on the production line. The genomes were closed using a hybrid assembly method with short and long sequencing reads.

## ANNOUNCEMENT

Salmonella spp. are commonly found in food products, primarily in the poultry industry in Thailand (1). Outbreaks of Salmonella not only affect exportations but increase production costs (2). Strict quality control, including the food safety and health policy from the European Commission for detecting Salmonella in poultry meat, has been applied to the production line. In this work, three Salmonella strains were isolated from chicken meat and skin collected from a chicken farm in Kaeng Khoi, Thailand, and grown on buffered peptone water (BPW) medium at 37°C for 24 h. Preliminary screening of antibiotic resistance using the Vitek 2 system (AST-GN97 test kit; bioMérieux, USA) revealed resistance to ampicillin. Therefore, the genome sequences of all three Salmonella strains were analyzed using Oxford Nanopore Technologies (ONT) and Illumina sequencers. Briefly, genomic DNA (gDNA) was extracted using ZymoBIOMICS DNA kits (Zymo Research, USA). For ONT, DNA library preparation was performed using the rapid barcoding kit (SQK-RBK004), prior to sequencing on a flow cell (vR9.4.1, FLO-MIN106) using the MinION Mk1c device for 48 h. Electrical signals from the sequencer were base called using the high accuracy model and demultiplexed using Guppy v5.0.7 (3), followed by adapter trimming using Porechop v0.2.4 (https://github.com/rrwick/Porechop). ONT reads of <500 bp were filtered using NanoFilt v2.6.0 (4) and quality checked using NanoPlot v1.28.1 (4). For Illumina, 100-bp paired-end libraries were prepared using the TruSeq Nano DNA kit and sequenced using the HiSeq 2500 sequencer (Illumina, USA). The raw reads were adapter trimmed using Skewer v0.2.2 (5).

Overall, ONT generated 30,725 (*N*_50_, 11,526 bp), 24,339 (*N*_50_, 10,638 bp), and 54,451 (*N*_50_, 11,393 bp) raw reads, while Illumina provided 2,797,747, 4,288,768, and 3,426,598 raw reads for strains SalSpp07, SalSpp08, and SalSpp09, respectively. Both sets of high-quality reads were then used for hybrid genome assembly using Unicycler v0.4.8 (6). The complete genome was determined using Bandage v0.9.0 (7). The genomic characteristics, including the genome size, number of contigs, and GC content (%), were examined using QUAST v5.0.2 (8). MOB-suite v3.0.3 was used for plasmid typing and to construct the complete genome (9). Taxonomic identification was performed using the Genome Taxonomy Database Toolkit (GTDB-Tk) v1.5.1 (10). Default parameters were used for all bioinformatics software. Genome annotation was performed using the NCBI Prokaryotic Genome Annotation Pipeline (PGAP) v4.11 (11). The genomic features of the three Salmonella isolates are summarized in Table 1. The antimicrobial resistance (AMR) genes were annotated using AMRFinderPlus v3.10.40 (12). Of these, *aadA2*, *aac(3)-IId*, *bla*_TEM-1_, and *lnu*(F) were found in both strains SalSpp07 and SalSpp09, with 99.6 to 100% identity. In addition, *tet*(A) (100% identity) was present in strain SalSpp07, and the D-to-Y change at position 87 encoded by *gyrA* [*gyrA*(D87Y)] (99.9% identity) was present in strain SalSpp08. In conclusion, all the Salmonella sp. genomes were closely related to S. enterica based on the average nucleotide identity (ANI) with the reference genome (GenBank accession number AE006468.2), with ANIs of 98 to 99%.

**TABLE 1:** Genomic characteristics of three Salmonella enterica strains isolated from poultry meat

Genomic feature	Data for strain:		
	SalSpp07	SalSpp08	SalSpp09
Size (bp)	5,125,631	4,854,412	4,966,490
GC content (%)	52.1	52.1	52.0
ANI (%)	99.79	98.2	98.5
No. of contigs	4	2	3
No. of CDSs^*a*^	4,834	4,550	4,639
No. of rRNAs	22	21	22
No. of tRNAs	85	82	87
GenBank accession no.			
Genome	CP104484	CP104482	CP104479
Plasmid(s)	CP104485, CP104486, CP104487	CP104483	CP104480, CP104481

a CDSs, coding DNA sequences.

### Data availability.

All complete genome sequences have been deposited at GenBank under the BioProject accession number PRJNA879268. The Illumina reads are available under the SRA accession numbers SRR21539848, SRR21539847, and SRR21539846. The ONT reads are available under SRR21539843, SRR21539842, and SRR21539841.
